# Real-world regorafenib use among patients with advanced gastrointestinal stromal tumor in the United States

**DOI:** 10.1371/journal.pone.0353357

**Published:** 2026-07-31

**Authors:** Ryan A. Denu, Sreevalsa Appukkuttan, Brian Hocum, Nick Liao, Arvind Katta, Jihaeng Heo, Bridgette Schroader, Risho Singh, Svetlana Babajanyan, Neeta Somaiah

**Affiliations:** 1 Sarcoma Medical Oncology, University of Texas MD Anderson Cancer Center, Houston, Texas, United States of America; 2 Bayer HealthCare Pharmaceuticals Inc, Whippany, New Jersey, United States of America; 3 Cencora, Conshohocken, Pennsylvania, United States of America; University Hospital Heidelberg, GERMANY

## Abstract

**Background:**

Gastrointestinal stromal tumors (GIST) are the most common gastrointestinal soft tissue sarcoma. Tyrosine kinase inhibitors are guideline-recommended therapy; however, resistance often occurs, requiring subsequent therapy. Regorafenib is a multikinase inhibitor approved for third-line therapy. Real-world data surrounding regorafenib’s use and place in therapy are limited.

**Objective:**

To understand real-world regorafenib utilization and patient characteristics among US patients with advanced GIST.

**Methods:**

This retrospective cohort claims analysis used data from Merative™ MarketScan^®^ research databases and included patients with ≥1 pharmacy claim for regorafenib during the identification period (10/2015–5/2023) and ≥1 GIST diagnoses any time prior to/on index date (first regorafenib prescription claim). Primary outcomes included duration of therapy (DOT) and time to next therapy (TTNT). Outcomes were stratified based on prior GIST treatment during the baseline period (BL) and initial regorafenib dose (i.e., low dose [LD] or regorafenib standard dose [RSD]) as of the index date.

**Results:**

Nearly half (45.2%) of patients received imatinib and sunitinib prior to regorafenib initiation, and 73.5% received RSD. Patients who received prior imatinib or sunitinib alone before regorafenib had a numerically longer median DOT with regorafenib than those who received both in the BL before regorafenib (142.5 days [IQR: 87–257.5] vs 95 days [IQR: 53–192]). Patients receiving LD and RSD demonstrated similar median DOT (103.0 days [IQR: 41.5, 210.5] vs 94.5 days [IQR: (35.0, 171.0]) and TTNT (143 days [IQR: 70–293] vs 141 days [IQR: 77–191]).

**Conclusions:**

Patients on LD and RSD had similar DOT and TTNT. Acknowledging the limitations from this real-world data, patients with prior imatinib or sunitinib alone appeared to have longer DOT on regorafenib than those who received both. Further research is warranted to explore the clinical benefits of these differences.

## Introduction

Gastrointestinal stromal tumors (GIST) are the most common gastrointestinal (GI) soft tissue sarcoma [1–3]. The incidence of GIST is approximately 4,000–6,000 cases per year in the United States (US) [4] and 10–15 cases per million worldwide [1,5]. Most tumors occur in the stomach and small intestine, and advanced-stage disease is found at diagnosis in 47% of cases, with metastases to the liver and peritoneum being the most common sites [6]. Although GIST can be surgically resected when identified early, approximately 40% of cases recur and/or metastasize, which may require multiple lines of systemic treatment [7].

The main oncogenic drivers for a majority of GISTs are mutated receptor tyrosine kinases. Thus, targeted therapy with tyrosine kinase inhibitors (TKIs) is the current standard of care in the treatment of advanced or metastatic GIST [8–11]. While these agents target a wide range of mutations for cancer treatment [12], in GIST patients, TKIs most commonly target *KIT* or *PDGFRA* gain-of-function mutations found in over 80% of GISTs [10,13]. Imatinib was the first TKI approved for GIST and remains the recommended first-line therapy for the treatment of unresectable, progressive, or metastatic GIST [9,11]. Although 85% to 90% of patients treated with imatinib obtain disease control [14], primary resistance is seen in 10% to 15% of patients [15] and 40% to 50% develop secondary resistance due to newly acquired mutations in *KIT* or *PDGFRA* [14–18], requiring second-line TKIs, such as sunitinib, and subsequent lines of therapy.

Regorafenib, a multikinase inhibitor, is indicated for previously-treated, locally advanced, unresectable or metastatic GIST [19]. Regorafenib is guideline-recommended as the preferred third-line therapy for patients with advanced or metastatic GIST that has progressed on imatinib and sunitinib [9]. Despite guideline recommendations as an established third-line therapy, anecdotal reports suggest regorafenib may be used in earlier lines of therapy in the real world [20].

However, regorafenib’s current use and potential place in therapy remain to be explored, as there is sparse data regarding its real-world utilization to treat GIST in US patients [21,22]. Reasons for the paucity of data could be the small patient pool indicated for regorafenib use following the failure of first- and second-line therapies and the lack of an International Classification of Diseases, 9th Revision, Clinical Modification (ICD-9-CM) diagnosis code specific to GIST prior to the implementation of ICD-10 in 2015 [23]. The lack of available utilization data limits the ability to determine practice patterns. To contribute to a better understanding of the real-world use of regorafenib in GIST, this study aimed to capture demographic and clinical characteristics as well as treatment utilization amongst patients with advanced or metastatic GIST treated with regorafenib in the US.

## Materials and methods

### Data source

This study was a retrospective cohort analysis of data from the Merative™ MarketScan^®^ Research Databases (MarketScan), specifically the Commercial Claims & Encounters and Medicare Supplemental & Coordination of Benefits databases. MarketScan is a large, national, deidentified claims database which tracks longitudinal inpatient and outpatient care and pharmacy claims, including commercial and Medicare insurance claims. The Medicare Supplemental & Coordination of Benefits database includes a subset of the Medicare population that are Medicare-eligible retirees with employer-sponsored Medicare Supplemental and Medicare Advantage plans. Data in this study were deidentified and fully anonymized in accordance with the Health Insurance Portability and Accountability Act prior to being accessed on March 27, 2024; therefore, neither institutional review board approval nor patient consent was required.

### Study design and cohort

**Fig 1:**
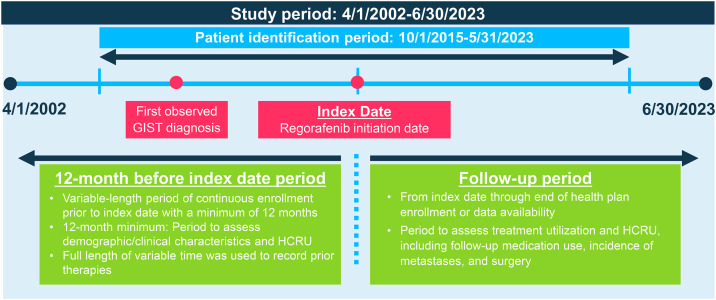
Study design. Key: GIST – gastrointestinal stromal tumor; HCRU – healthcare resource utilization.

The study period was from the beginning of database availability (April 1, 2002) through June 3, 2023 (Fig 1). Patients were included in the analysis if they had ≥ 1 pharmacy claim for regorafenib during the identification period from October 1, 2015, through May 31, 2023. The first prescription claim date for regorafenib during the identification period was designated as the index date. Patients were also required to have ≥1 medical claim with GIST diagnosis codes, defined by ICD-10-CM diagnosis codes, any time prior to or on the index date. The 12-month before index date period was defined as a variable period of ≥12 months prior to, and up to, the index date. Additionally, patients needed to have continuous health plan enrollment ≥12 months prior to the index date (exclusive), and ≥28 days after the index date (inclusive). Patients were excluded if they were <18 years of age as of the index date. The follow-up period was defined as the time between the index date through the earliest of disenrollment or end of data availability, which varied in length by patient.

### Study variables and outcomes

Demographics, clinical characteristics, medication use, and history of malignancy data were collected for patients included in the study. Demographic variables, including age, sex, US geographic region, payer type, and health plan type, were measured on the index date. To understand the characteristics of patients being prescribed regorafenib, clinical characteristics were measured during the 12-month before index date period, which included the Quan-Charlson Comorbidity Index (CCI) score [24,25], surgery, genetic testing, and metastases. Medications and history of malignancy prior to GIST diagnosis were collected throughout the entire 12-month before index date period.

Primary outcomes were duration of therapy (DOT) and time to next therapy (TTNT). DOT was defined as the length of time from the date of the first claim for regorafenib to either the date of a claim for a new GIST therapy or discontinuation of regorafenib, whichever came earlier. Discontinuation was defined as ≥60-day gap in claims data. TTNT was defined as the interval between the index date and the date of initiation of the next GIST therapy. Secondary outcomes were measured during the study period and included follow-up medication use and surgery. Outcomes were stratified by subgroups during the 12-month before index date period based on prior treatment for GIST with commonly prescribed therapies in the real world to ascertain regorafenib’s place in therapy. Subgroups of interest included imatinib only or sunitinib only prior to regorafenib, pazopanib only prior to regorafenib, imatinib and sunitinib prior to regorafenib, ripretinib only prior to regorafenib, or avapritinib only prior to regorafenib. Note, because GIST is associated with a long history, these prior therapies may not represent all therapy exposures throughout a patient’s disease course. Groups were also stratified by initial regorafenib dose into low dose (LD) regorafenib or standard dose regorafenib cohorts (RSD) as of the index date. LD was defined as <160 mg per day over 21 days in a 28-day cycle and RSD was defined as an average daily dose of 160 mg per day for 21 days in a 28-day cycle.

### Data analysis

Descriptive statistics were used to analyze all study outcomes (i.e., mean, median, etc). No direct comparisons were made between subgroups using statistical tests. Mean values were presented with standard deviation (SD) and median values were presented with interquartile range (IQR). In addition to descriptive analyses, time-to-event analyses were performed using Kaplan-Meier curves to depict DOT and TTNT. The event analyzed for DOT was defined as the date of a claim for a new GIST therapy or regorafenib discontinuation. Patients who experienced disenrollment or entered the end of the observation period while on regorafenib were censored. For TTNT, the date of initiation of the next GIST therapy was the analyzed event and patients without subsequent therapy were censored. Log-rank tests were performed to compare Kaplan-Meier curves amongst study groups. All analyses were conducted using the software package SAS version 9.4 (SAS Institute Inc., Cary, NC, USA).

### Sensitivity analysis

A sensitivity analysis was performed using a more comprehensive approach to patient selection to account for the potential of missed or delayed documentation of a GIST ICD code. The analysis reported the number of patients who received regorafenib during the study identification period irrespective of when they were diagnosed with GIST (i.e., their GIST diagnosis could have been documented after the index date).

## Results

**Fig 2:**
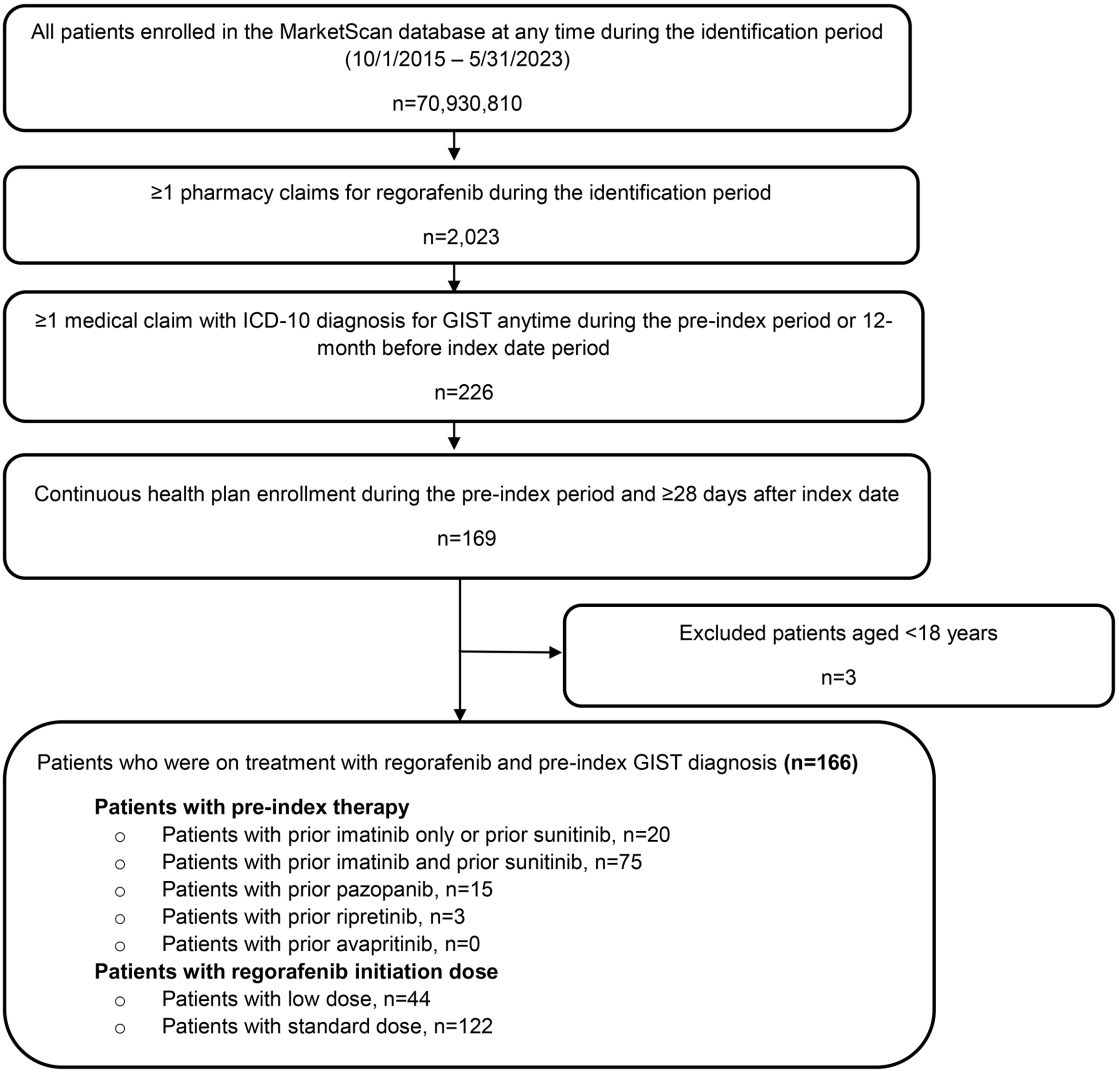
Patient selection. Key: GIST – gastrointestinal stromal tumor.

Among 70,930,810 patients enrolled in the MarketScan database at any time during the identification period (10/1/2015–5/31/2023), 166 patients met the inclusion criteria of a pre-index GIST diagnosis and receipt of regorafenib (Fig 2). The mean age was 57.6 years (SD: 15.2), 57.2% were male, and 73.5% had commercial insurance. When categorized by prior treatment for GIST, 20 (12.0%) patients had received prior imatinib (n = 8) or sunitinib only (n = 12), 75 (45.2%) had received imatinib and sunitinib, 15 (9.0%) had received prior pazopanib only, and 3 (1.8%) had received ripretinib only. When categorized by regorafenib dose, 44 (26.5%) patients received LD and 122 (73.5%) received RSD. Additional baseline characteristics for the overall population and each of the subgroups are presented in Table 1.

**Table 1:** Baseline characteristics.

Characteristics	All patients N = 166	Imatinib only/ Sunitinib only prior to regorafenib n = 20	Pazopanib only prior to regorafenib n = 15	Imatinib+ Sunitinib prior to regorafenib n = 75	Low dose regorafenib n = 44	Standard dose regorafenib n = 122
Measured as of index date						
Mean age, years (SD)	57.6 (15.2)	60.9 (14.0)	48.0 (13.9)	63.2 (11.8)	62.4 (15.3)	55.9 (14.9)
Male, n (%)	95 (57.2)	13 (65.0)	6 (40.0)	46 (61.3)	28 (63.6)	67 (54.9)
Region, n (%)						
North-central	40 (24.1)	2 (10.0)	4 (26.7)	20 (26.7)	14 (31.8)	26 (21.3)
Northeast	32 (19.3)	7 (35.0)	4 (26.7)	12 (16.0)	6 (13.6)	26 (21.3)
South	71 (42.8)	6 (30.0)	6 (40.0)	34 (45.3)	16 (36.4)	55 (45.1)
West	23 (13.9)	5 (25.0)	1 (6.7)	9 (12.0)	8 (18.2)	15 (12.3)
Payer type, n (%)						
Medical (basic or major)	113 (68.1)	12 (60.0)	15 (100.0)	44 (58.7)	23 (52.3)	90 (73.8)
Comprehensive	9 (5.4)	2 (10.0)	0 (0.0)	4 (5.3)	3 (6.8)	6 (4.9)
EPO	34 (20.5)	4 (20.0)	0 (0.0)	22 (29.3)	13 (29.5)	21 (17.2)
HMO	10 (6.0)	2 (10.0)	0 (0.0)	5 (6.7)	5 (11.4)	5 (4.1)
Insurance type, n (%)						
Commercial	122 (73.5)	14 (70.0)	15 (100.0)	48 (64.0)	26 (59.1)	96 (78.7)
Medicare	44 (26.5)	6 (30.0)	0 (0.0)	27 (36.0)	18 (40.9)	26 (21.3)
Index year						
2015	12 (7.2)	2 (10.0)	2 (13.3)	9 (12.0)	3 (6.8)	9 (7.4)
2016	11 (6.6)	2 (10.0)	2 (13.3)	6 (8.0)	2 (4.5)	9 (7.4)
2017	17 (10.2)	2 (10.0)	0 (0.0)	13 (17.3)	3 (6.8)	14 (11.5)
2018	18 (10.8)	3 (15.0)	1 (6.7)	9 (12.0)	2 (4.5)	16 (13.1)
2019	30 (18.1)	4 (20.0)	2 (13.3)	11 (14.7)	5 (11.4)	25 (20.5)
2020	24 (14.5)	2 (10.0)	3 (20.0)	8 (10.7)	10 (22.7)	14 (11.5)
2021	24 (14.5)	3 (15.0)	4 (26.7)	6 (8.0)	7 (15.9)	17 (13.9)
2022	19 (11.4)	2 (10.0)	0 (0.0)	9 (12.0)	9 (20.5)	10 (8.2)
2023	11 (6.6)	0 (0.0)	1 (6.7)	4 (5.3)	3 (6.8)	8 (6.6)
Time before index date, months (median; IQR)	81.0 (58.7; 26.6, 128.0)	75.9 (28.3; 16.3, 155.5)	95.6 (92.2; 47.4, 134.3)	87.4 (59.6; 34.1, 131.0)	79.5 (56.7; 26.4, 131.2)	81.6 (58.8; 26.6, 126.3)
Measured during 12-month before index date period						
Mean CCI^a^ (SD)	8.6 (2.6)	7.5 (3.1)	9.3 (1.7)	8.3 (2.9)	8.8 (2.3)	8.5 (2.7)
CCI^a^ categories						
0-8	71 (42.8)	11 (55.0)	7 (46.7)	35 (46.7)	18 (40.9)	53 (43.4)
9-10	63 (38.0)	7 (35.0)	5 (33.3)	25 (33.3)	14 (31.8)	49 (40.2)
≥ 11	31 (18.7)	2 (10.0)	3 (20.0)	15 (20.0)	11 (25.0)	20 (16.4)
Reported presence of metastases, n (%)	148 (89.2)	15 (75.0)	15 (100.0)	65 (86.7)	41 (93.2)	107 (87.7)
Measured at any point before index date period						
Baseline medications, n (%)						
Imatinib	83 (50.0)	8 (40.0)	2 (13.3)	75 (100.0)	18 (40.9)	65 (53.3)
Pazopanib	15 (9.0)	2 (10.0)	15 (100.0)	2 (2.7)	3 (6.8)	12 (9.8)
Sorafenib	6 (3.6)	2 (10.0)	1 (6.7)	1 (1.3)	2 (4.5)	4 (3.3)
Sunitinib	87 (52.4)	12 (60.0)	4 (26.7)	75 (100.0)	21 (47.7)	66 (54.1)
Other^b^	8 (4.8)	1 (5.0)	1 (6.7)	5 (6.7)	1 (2.3)	7 (5.7)

Note: Baseline demographics for subgroups with n < 10 are not presented as statistics from such low patient count subgroups are not expected to be reliable.

^a^The CCI is a weighted index utilizing the presence of comorbid conditions to predict the risk of death within 10 years. Lower scores indicate fewer serious comorbidities. Categories were chosen to represent common cutoffs that would impact treatment decisions.

^b^Other medications included dasatinib, everolimus, larotrectinib, nilotinib, and ripretinib.

Key: CCI – Charlson comorbidity index; EPO – employer provider organization; GIST – gastro-intestinal stromal tumor; HMO – health maintenance organization; SD – standard deviation.

**Fig 3:**
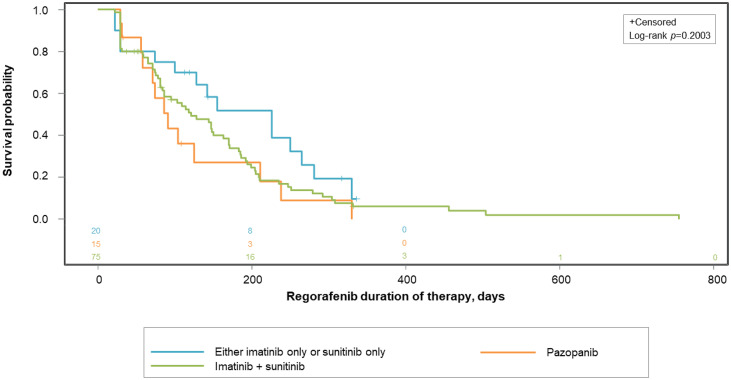
Kaplan-Meier plot of DOT by prior treatment. ^a^ *P*-value was calculated via one-sided log-rank test. Key: DOT – duration of therapy.

**Fig 4:**
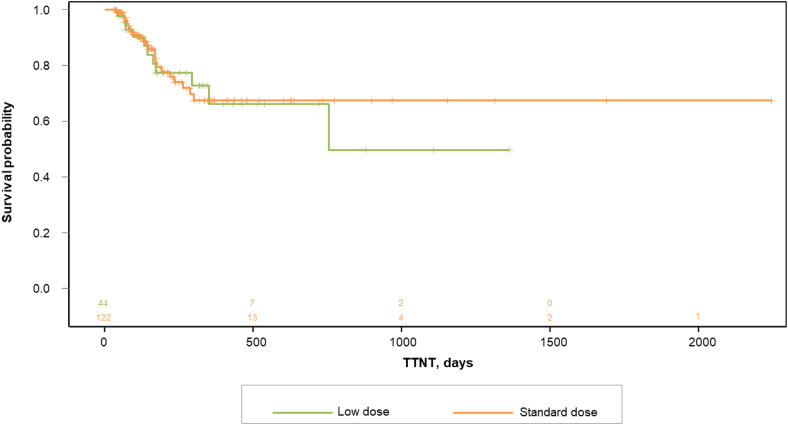
Kaplan-Meier plot of TTNT: regorafenib LD vs RSD. ^a^ *P*-value was calculated via one-sided log-rank test. Key: LD – low dose; RSD – regorafenib standard dose; TTNT – time to next treatment.

After a median follow-up of 221.5 days (IQR:109.0, 416.0), the median regorafenib DOT was 95.0 days (IQR: 36.0, 177.0). Thirty-four patients (20.5%) switched therapy, with a median TTNT of 142.0 days (IQR: 77.0, 191.0). Patients with prior imatinib or sunitinib alone (n = 20) had a numerically longer median DOT than those who received both (n = 75) in the 12-month before index date period (142.5 days [IQR: 87.0, 257.5] vs 95.0 days [IQR: 53.0, 192.0]) (Fig 3). No meaningful differences were observed in median DOT between patients on LD regorafenib (n = 44)and those on RSD (n = 122) (103.0 days [IQR: 41.5, 210.5] vs 94.5 days [IQR: (35.0, 171.0]). Additionally, both groups had similar median TTNT (143.0 days [IQR: 70.0, 293.0] vs 141.0 days [IQR: 77.0, 191.0]) (Fig 4).

Medications prescribed during the follow-up period were ripretinib (12.7%), imatinib (10.8%), sunitinib (6.0%), cabozantinib (3.0%), nilotinib (2.4%), dasatinib, sorafenib, pazopanib (1.8% each), and everolimus (1.2%).

### Sensitivity analysis

A total of 190 patients were included in the sensitivity analysis. The baseline characteristics were similar to those observed in the main analysis. The median regorafenib DOT was 103.5 (IQR: 37.0, 186) and 38 patients (20.0%) switched therapy, with a median TTNT of 154.0 days (IQR: 81.0, 234.0). Patients with prior imatinib or sunitinib alone (n = 26) in the 12-month before index date period had a numerically longer median regorafenib DOT than those who received both imatinib and sunitinib (n = 80) during the 12-month before index date period (149.0 days [IQR: 100.0, 281.0] vs 110.5 days [IQR: 58.5, 201.5]). Note, the 12-month before index date period was a variable period that included at least 12 months prior to and up to the index date. Patients on LD regorafenib (n = 50) had similar median DOT to those on RSD (n = 140) (103.0 days vs 103.5 days). Median TTNT was 152.5 days (IQR: 82.0, 320.5) for the LD cohort and 157.0 days (IQR: 81.0, 219.0) for the RSD cohort.

## Discussion

In this retrospective cohort analysis, patients who received regorafenib after imatinib or sunitinib demonstrated a numerically longer DOT than those who received regorafenib after both imatinib and sunitinib. While numerically different, no clinically meaningful differences were observed in DOT or TTNT between patients on LD regorafenib and those on RSD. The sensitivity analysis was consistent with our main results for prior therapy subgroup DOT but had slightly different LD vs RSD results, with similar median DOT for those receiving LD and RSD regorafenib and a shorter TTNT in those receiving LD regorafenib. Overall, differences in DOT reflect prescribing patterns, patient selection, and administrative factors but may not accurately reflect treatment effectiveness, as it is heavily confounded by physician and patient preference, toxicity management strategies, patient access, and planned treatment breaks.

To our knowledge, this is the first study that analyzes the real-world utilization of regorafenib in lines of therapy earlier than approved for treatment of advanced or metastatic GIST in the US. In this analysis, prior to regorafenib initiation, 45.2% of patients received imatinib and sunitinib and 12% received imatinib or sunitinib only, showing that regorafenib is perhaps being used as an earlier line of therapy in certain situations despite its approved indication and guideline recommendation as third-line therapy. One caveat to this study is the lack of captured use of treatments through clinical trials or patient assistance programs, which are inherent limitations in claims-based studies and limit applicability of results.

The earlier use of regorafenib may be due to physician preference or patient- or market-specific drivers, such as mutation profiles [26], or potential inaccessibility of sunitinib for some patients, as it is now off patent and does not have any available financial assistance programs [27]. These drivers are not captured in claims data but are important to understand. DOT was numerically longer in patients who received regorafenib after imatinib or sunitinib only compared to those who received both imatinib and sunitinib; there are multiple potential drivers of longer DOT with earlier use of regorafenib such as specific GIST molecular profiles and improved patient tolerance prior to multiple lines of therapy. Similarly, there may be drivers of shorter DOT in those who received regorafenib after two therapies such as a more aggressive or unresponsive disease profile.

Further, analysis of regorafenib initial dose showed that 73.5% received RSD while 26.5% received LD, which demonstrated an additional discrepancy between real-world and guideline-recommended use of regorafenib. Median DOT and TTNT were similar between patients who received LD vs those who received RSD. While limited by several factors noted below, these results help portray the real-world treatment patterns and characteristics of a patient population with advanced metastatic GIST.

Prior to our study, two real-world evidence analyses were performed in the US to investigate regorafenib utilization in clinical practice. According to an observational retrospective study conducted by Call et al [21], regorafenib was used off-label in earlier lines of therapy, however, outcomes assessing clinical efficacy of second-line use (i.e., overall survival, progression free survival, etc.) were not analyzed due to small sample size. Among patients with advanced GIST who received single agent regorafenib (n = 111), regorafenib was used second-line in 1.8% (n = 2) of patients, third-line in 50.5% (n = 56), and fourth-line in 21.6% (n = 24) [21]. It is important to note that since regorafenib was approved for third-line therapy for GIST in 2013 [28], these rates are likely to be lower than more recent real-world regorafenib utilization rates in each line of therapy. In another retrospective analysis conducted by Schvartsman et al [22], among a small group of patients (n = 28) at a single institution, the majority of clinicians started patients on regorafenib 120 mg continuous regimen rather than intermittent RSD, with 79% of patients receiving 120 mg. While our study was unable to ascertain if any patients were utilizing continuous dosing regimens due to the claims data source, these real-world reports highlight that there are multiple alternative regorafenib dosing regimens being utilized in real-world settings for patients with advanced or metastatic GIST and further underscore the importance of reporting real-world data to assess utilization of regorafenib in this patient population.

### Limitations

Inherent limitations of real-world studies which utilize large claims databases include miscoding, data errors, and missing data. There is limited generalizability of our results due to limitations of claims data and number of patients. Only commercially insured or Medicare patients were evaluated, thus, information on populations such as those who were uninsured, self-insured, or insured under Veterans Affairs or Medicaid were missing. Further, excluding patients who lacked continuous coverage 12 or more months or had a different commercial insurance plan before regorafenib initiation may have led to the non-capture of all prior therapies. Additionally, this analysis only included ICD-10-CM GIST-specific codes, as there were previously no ICD-9-CM codes specific to GIST diagnosis. Procedure codes for genetic testing were available, but no testing results were available in the claims data therefore they could not be included in the study.

Due to the long natural history of the disease, the actual time of first diagnosis of GIST and all therapies may not have been captured, which may have led to underrepresentation of treatment utilization; therefore, the 12 patients who received prior sunitinib alone and 15 patients who received prior pazopanib alone could be due to prior therapies being utilized that were not captured due to the study time frame or therapies being utilized that were not captured in the claims data. For example, the finding that 12 of the patients who had received prior sunitinib alone may represent patients who received imatinib in the adjuvant setting, which would likely not have been captured in this analysis. Additionally, treatments received in clinical trials or through patient assistance programs were not captured in the claims data. The dosing cohorts were defined based on pills dispensed without access to information on whether the patients took the pills. Pill counts were based on physician prescribing patterns and may not have always reflected the actual starting dose.

## Conclusions

While acknowledging the limitations of a claims-based analysis, this real-world study reveals that the utilization patterns of regorafenib may not always align with guideline recommendations, potentially reflecting use in earlier lines of therapy for a small subset and different dosing. Patients on LD regorafenib had DOT and TTNT similar to those on RSD. Regorafenib initiated after imatinib or sunitinib alone compared to after both agents showed a numerically longer DOT. However, additional studies will be needed to clarify benefits in earlier therapy lines and, as newer treatments emerge, to understand responses across molecular profiles so that the most effective drugs can be sequenced earlier to match each patient’s profile. Despite the noted limitations, this is, to our knowledge, the first study that captures the real-world utilization of regorafenib in advanced or metastatic GIST in a US population. Findings from this study help portray the real-world treatment patterns and characteristics of a patient population with advanced or metastatic disease.
