# Supplementary material for: Real-world regorafenib use among patients with advanced gastrointestinal stromal tumor in the United States
Source: PLoS One. 2026 Jul 31;21(7):e0353357. doi: 10.1371/journal.pone.0353357 (PMC13426992; doi:10.1371/journal.pone.0353357)
Supplement: Attachment — Submitted filename: Journal revision table_18MAR26_Final.docx [file pone.0353357.s002.docx]

**Journal feedback**

| **Comment** | **Recommended action** |
| --- | --- |
| **Reviewer #1** |  |
| Clinical inferences exceed what the data can support, and there are methodologic, interpretive, and presentation issues that substantially limit impact in its current form.  The manuscript would benefit from tightening its objectives, clarifying what can and cannot be concluded, and reducing causal language. As written, it risks overstating practice-changing implications from descriptive claims data.  Claims-based DOT and TTNT are weak surrogates for clinical benefit and should not be framed as efficacy proxies. | Thank you for your comments. By addressing the edits and comments from both reviewers, we believe we have addressed this comment. |
| Reframe the study explicitly as a treatment utilization and sequencing analysis, not an outcomes study. Avoid language implying benefit, tolerance, or superiority.  Overinterpretation of DOT and TTNT: DOT and TTNT are heavily confounded by: o Physician preference o Toxicity management strategies o Access and insurance o Planned treatment breaks  Statements suggesting longer DOT reflects better tolerance or response are speculative and unsupported.  Replace with neutral phrasing: “Differences in DOT may reflect prescribing patterns, patient selection, or administrative factors rather than treatment effectiveness. | We have updated the manuscript to include these limitations within the discussion section, limitations section, and conclusions section. |
| Line of therapy classification is fundamentally incomplete and not reflect access to drugs through patient assistance programs.  This limitation should be moved from a minor limitation to a central interpretive caveat and reiterated in the Discussion conclusions. | We have added the following sentence to the first paragraph in the discussion:  One caveat to this study is the lack of captured use of treatments through clinical trials or patient assistance programs, which are inherent limitations in claims-based studies and limit applicability of results.  We have added the following sentence in the limitations: Additionally, treatments received in clinical trials or through patient assistance programs were not captured in the claims data.  Updated relevant conclusion sentences as follows:  While acknowledging the limitations of a claims-based analysis, this real-world study reveals that the utilization patterns of regorafenib may not always align with guideline recommendations, potentially reflecting use in earlier lines of therapy for a small subset and different dosing.  Despite the noted limitations, this is, to our knowledge, the first study that captures the real-world utilization of regorafenib in advanced or metastatic GIST in a US population. |
| The following statements are overreaching:  Despite guideline recommendations as an established third-line therapy, regorafenib may be used in earlier lines in the real world, perhaps for its ability to target a wide range of cancer pathways.- there is no data to substantiate this claim for the real world- and this may be misleading.  Discussion statement- The earlier use of regorafenib may be due to physician preference or patient- or market-specific drivers, such as mutation profiles, including KIT activation loop mutations (exon 17 or 18) or SDH deficient GIST which are more responsive to regorafenib and resistant to sunitinib- is completely unfounded with no data. | Updated both statements:  Despite guideline recommendations as an established third-line therapy, anecdotal reports suggest regorafenib may be used in earlier lines of therapy in the real world.[20]  The earlier use of regorafenib may be due to physician preference or patient- or market-specific drivers, such as mutation profiles,[26] or potential inaccessibility of sunitinib for some patients, as it is now off patent and does not have any available financial assistance programs.[27] |
| Does this comment truly represent the time period of the data or is this relevant to today?- potential inaccessibility of sunitinib for some patients, as it is now off patent and does not have any available financial assistance programs. | We would like to clarify that this statement is meant to align with the study’s observation period, which includes data through 2023, and is not intended to reflect an older era of practice.  We also recognize that access dynamics can evolve over time. While sunitinib is now off patent, some patients may still experience access barriers (e.g., coverage variability, formulary restrictions, or limited assistance options for certain products), which may affect real-world availability. In that context, we felt it was appropriate to note that regorafenib could represent an option to help address potential unmet need in earlier lines of care when access to sunitinib is challenging. |
| **Reviewer #2** |  |
| Line 88-89: "This lack of available data highlights a potential practice gap". This sentence is unclear. Perhaps the authors meant to say "The lack of available data limits our ability to determine practice patterns," or similar? | Agreed, updated sentence: Thes lack of available data limits the ability to determine practice patterns. |
| Line 107: Note "error" RE reference. Several other instances in manuscript. | Thank you for letting us know. The navigation does not show those errors in our copy, but we removed navigation links anyway assuming the formatting will be changed for final publication, so they aren’t needed. |
| Line 128: CCI score; to simplify for the reader, could you add what the categories mean by including something like: "Briefly, lower CCI score indicates fewer serious comorbidities..." and consider specifying why you split into cohorts of score 2-3 vs score 4+ (from table 1). | We have added the following footnote to Table 1: The CCI is a weighted index utilizing the presence of comorbid conditions to predict the risk of death. Lower scores indicate fewer serious comorbidities. Categories were chosen to represent common cutoffs that would impact treatment decisions.  Additionally, the CCI group summary has been updated to a more balanced grouping center around the median. |
| Line 138-139: Table 1 subgroups and stratification should line up with the categories listed here. Rather than putting "ie" I suggest detailing exactly what your subgroups are. Keep this consistent with text and figures. In the table for example, "pazopanib" is a header and it is not clear if this means pazopanib only vs any exposure to pazopanib. Further, since ripretinib was FDA approved during the study period (and since it is also an activation loop inhibitor, I suggest adding it as a subgroup. I also noted that sorafenib is listed in table 1, but here, avapritinib is listed. | We have updated the text to clearly state the subgroups and removed use of ie. We have also updated the headings of table 1 to match the text.  We have added the following footnote to Table 1:  Note: Baseline demographics for subgroups with n<10 are not presented as statistics from such low patient count subgroups are not expected to be reliable. |
| Finally, a big concern about these data is that many of the patients included in the study may have accessed TKI with patient assistance programs or as participants on clinical trials. I assume these data cannot capture this. Perhaps one sensitivity analysis could exclude any patients with a >3 month gap in therapies since we know that most patients with GIST are continuously on TKI therapy. This would hopefully remove any patients who were exposed to unknown TKI therapies. | Unfortunately, we are unable to complete additional analyses at this time.  We have added the following sentence to the first paragraph in the discussion:  One caveat to this study is the lack of captured use of treatments through clinical trials or patient assistance programs, which are inherent limitations in claims-based studies and limit applicability of results.  We have also added the following sentence in the limitations: Additionally, treatments received in clinical trials or through patient assistance programs were not captured in the claims data. |
| Line 142: "Groups were also stratified by initial regorafenib dose..." Some clinicians start TKI at a lower dose and escalate as tolerated, and the initial RX may reflect this strategy. Would it be possible to analyze the dose that the patient received most during their time on treatment (acknowledging that some may be at a lower dose due to toxicity). | This is an interesting perspective and we agree. This still highlights a difference from dose per label. Unfortunately, calculating dose received during entire treatment duration is outside the scope of our current paper. |
| Line 172: how were the remaining cases categorized (the ones included do not add up to 100%)? | The text was updated to specify subgroups of interest are reported and a footnote has been added under Table 1 to explain which subgroups are reported. |
| Table 1: typo in header "measured at any point in before index date period". Remove "in"? | Updated, thank you |
| Line 187: include “n” for each cohort in body of text (prior IM or SU vs IM+SU) | Added n’s |
| Line 190: Sentence fragment, please revise. Also include “n” for each cohort (LD vs RSD). | Added n’s |
| Line 205-206: Without clinical context, I do not think including that 3 patients underwent surgery adds any information. Consider removing. | Removed sentence |
| Line 211-216: include “n” for each cohort (prior IM or SU vs IM+SU; LD vs RSD). | Added n’s |
| Line 217: Remove “slightly shorter”. These numbers are essentially the same so I suggest leaving out that description. You can just report the median TTNT for each cohort. | Updated to suggested text. |
| Line 221-226: The opening sentence is correct- although I still question whether it is clinically meaningful- but the second sentence RE LD vs RSD is overstated in my opinion. 103 vs 94.5 days are so similar and to highlight that patients on LD have longer DOT seems misleading. | We have updated the text to the following: While numerically different, no clinically meaningful differences were observed in DOT or TTNT between patients on LD regorafenib and those on RSD. |
| Line 239-242: I suggest commenting on whether result is statistically significant and clinically meaningful to warrant additional evaluation. Further, therapies used in earlier lines often have better clinical outcomes than when used in later lines, yet they may not be better than the approved earlier-line therapies. | We have softened this language as follows: DOT was numerically longer in patients who received regorafenib after imatinib or sunitinib only compared to those who received both imatinib and sunitinib; there are multiple potential drivers of longer DOT with earlier use of regorafenib such as specific GIST molecular profiles and improved patient tolerance prior to multiple lines of therapy. Similarly, there may be drivers of shorter DOT in those who received regorafenib after two therapies such as a more aggressive or unresponsive disease profile. |
| Line 245-246: Again, I suggest softening language here and mentioning that difference is similar and not clinically meaningful (1.5 weeks different). | Edited to: Median DOT and TTNT were similar between patients who received LD vs those who received RSD. |
| Line 248: I did not see underrepresented status indicated in Table 1. Please define what is meant here. | Edited to “underrepresented” as follows: “While limited by several factors noted below, these results help portray the real-world treatment patterns and characteristics of a patient population with advanced metastatic GIST. |
| Line 278-288: You may also mention that treatments received on clinical trials are not captured in these data. | Added sentence “Additionally, treatments received in clinical trials were not captured in the claims data.” |
| Line 290-291: I think given the limitations of this data type, it is challenging to make any conclusions about utilization patterns. | Updated line to soften this sentence: While acknowledging its limitations, this real-world study reveals that the utilization patterns of regorafenib may not always align with guideline recommendations, potentially reflecting use in earlier lines of therapy for a small subset and different dosing. |
| Line 292-294: As mentioned many times, 1.5 weeks difference should not be highlighted as a true difference. Please revise. | Revised to phrase DOT as similar instead of different: ”Patients on LD regorafenib had DOT and TTNT similar to those on RSD.” |
| Line 294-295: I’d suggest including more nuance if there is really a case for additional studies. Do you suggest a randomized trial evaluating regorafenib vs sunitinib, or bezuclastinib/sunitinib in 2nd line? Or regorafenib vs ripretinib for activation loop secondary mutations? This does not seem feasible and I’m not sure what additional studies should be done here. | Revised to: However, additional studies will be needed to clarify benefits in earlier therapy lines and, as newer treatments emerge, to understand responses across molecular profiles so that the most effective drugs can be sequenced earlier to match each patient’s profile. |
| Line 295-297: be sure to include US population; studies exist in other countries. | Added “US population” at the end of the sentence:  To our knowledge, this is the first study that captures the real-world utilization of regorafenib in advanced or metastatic GIST in a US population. |
| Line 297-298: I disagree with the conclusion that this adds nuance to the understanding of regorafenib treatment patterns and clinical outcomes. I am also not sure what “insufficiently characterized patient population” means. | We have updated the final sentence to state: Findings from this study help portray the real-world treatment patterns and characteristics of a patient population with advanced or metastatic disease. |
