# Supplementary material for: Real-world regorafenib use among patients with advanced gastrointestinal stromal tumor in the United States
Source: PLoS One. 2026 Jul 31;21(7):e0353357. doi: 10.1371/journal.pone.0353357 (PMC13426992; doi:10.1371/journal.pone.0353357)
Supplement: Attachment — Submitted filename: Response to Reviews 4.30.26.docx [file pone.0353357.s003.docx]

Response to Reviews

Reviewer #1: All comments appreciated. No further recommendations.

Appreciate the time and effort to make the changes.

*We thank the reviewer for their positive feedback and effort to help make our manuscript suitable for publication.*

Reviewer #2: Please update the abstract, (line 59) first sentence in the conclusion to conclude that TTNT *and* DOT were similar for LD and RSD.

*This has been corrected and now reads: “Patients on LD and RSD had similar DOT and TTNT.”*

Please correct the sentence in the abstract (line 54): "Patients who received prior imatinib or sunitinib alone
54 before regorafenib reported a numerically longer median DOT". ...The patients did not "report". You can say, for example, "The data showed that patients who received...had a numerically longer median DOT."

*This has been corrected and now reads: “Patients who received prior imatinib or sunitinib alone before regorafenib had a numerically longer median DOT…”*

Please the language for the sentence in the abstract (line 56): "LD demonstrated a longer median DOT compared to RSD" and instead indicate that they are similar.

*This has been corrected and now reads: “Patients receiving LD and RSD demonstrated similar median DOT…and TTNT…”*
