# Supplementary material for: Real-world regorafenib use among patients with advanced gastrointestinal stromal tumor in the United States
Source: PLoS One. 2026 Jul 31;21(7):e0353357. doi: 10.1371/journal.pone.0353357 (PMC13426992; doi:10.1371/journal.pone.0353357)
Supplement: Peer review history

# Peer review: Decision Letter 0

**DOI:** 10.1371/journal.pone.0353357.r001

**Authors:** Anette Duensing

16 Feb 2026

PONE-D-25-61423

Real-world regorafenib use among patients with advanced gastrointestinal stromal tumor in the United States

PLOS One

Dear Dr. Denu,

Thank you for submitting your manuscript to PLOS ONE. After careful consideration, we feel that your study has some merit but both reviewers indicate that there are significant flaws that need to be addressed before a final decision can be made.

Specifically, both reviewers felt strongly that that the authors overstated their findings. Furthermore, some questions about methodology, clinical meaningfulness and overall presentation were raised. Please address all of these points in a revised version of the manuscript.

Please submit your revised manuscript by Apr 02 2026 11:59PM. If you will need more time than this to complete your revisions, please reply to this message or contact the journal office at plosone@plos.org. When you're ready to submit your revision, log on to https://www.editorialmanager.com/pone/ and select the 'Submissions Needing Revision' folder to locate your manuscript file.

Please include the following items when submitting your revised manuscript:

- A letter that responds to each point raised by the academic editor and reviewer(s). You should upload this letter as a separate file labeled 'Response to Reviewers'.
- A marked-up copy of your manuscript that highlights changes made to the original version. You should upload this as a separate file labeled 'Revised Manuscript with Track Changes'.
- An unmarked version of your revised paper without tracked changes. You should upload this as a separate file labeled 'Manuscript'.

If you would like to make changes to your financial disclosure, please include your updated statement in your cover letter. Guidelines for resubmitting your figure files are available below the reviewer comments at the end of this letter.

If applicable, we recommend that you deposit your laboratory protocols in protocols.io to enhance the reproducibility of your results. Protocols.io assigns your protocol its own identifier (DOI) so that it can be cited independently in the future. For instructions see: https://journals.plos.org/plosone/s/submission-guidelines#loc-laboratory-protocols. Additionally, PLOS ONE offers an option for publishing peer-reviewed Lab Protocol articles, which describe protocols hosted on protocols.io. Read more information on sharing protocols at https://plos.org/protocols?utm_medium=editorial-email&utm_source=authorletters&utm_campaign=protocols.

We look forward to receiving your revised manuscript.

Kind regards,

Anette Duensing, M.D.

Academic Editor

PLOS One

Journal Requirements:

When submitting your revision, we need you to address these additional requirements.

1. Please ensure that your manuscript meets PLOS ONE's style requirements, including those for file naming. The PLOS ONE style templates can be found at

https://journals.plos.org/plosone/s/file?id=wjVg/PLOSOne_formatting_sample_main_body.pdf and

https://journals.plos.org/plosone/s/file?id=ba62/PLOSOne_formatting_sample_title_authors_affiliations.pdf.

2. Thank you for providing the following Funding Statement:

“This work was funded by Bayer HealthCare Pharmaceuticals, Inc. Notwithstanding those employed by Bayer, authors were not paid to participate. The funders, other than the named authors, did not play a role in study design, data collection and analysis, decision to publish, or preparation of the manuscript.”

We note that one or more of the authors is affiliated with the funding organization, indicating the funder may have had some role in the design, data collection, analysis or preparation of your manuscript for publication; in other words, the funder played an indirect role through the participation of the co-authors.

If the funding organization did not play a role in the study design, data collection and analysis, decision to publish, or preparation of the manuscript and only provided financial support in the form of authors' salaries and/or research materials, please review your statements relating to the author contributions, and ensure you have specifically and accurately indicated the role(s) that these authors had in your study in the Author Contributions section of the online submission form. Please make any necessary amendments directly within this section of the online submission form.  Please also update your Funding Statement to include the following statement: “The funder provided support in the form of salaries for authors [insert relevant initials], but did not have any additional role in the study design, data collection and analysis, decision to publish, or preparation of the manuscript. The specific roles of these authors are articulated in the ‘author contributions’ section.”

If the funding organization did have an additional role, please state and explain that role within your Funding Statement.

Please also provide an updated Competing Interests Statement declaring this commercial affiliation along with any other relevant declarations relating to employment, consultancy, patents, products in development, or marketed products, etc.

Within your Competing Interests Statement, please confirm that this commercial affiliation does not alter your adherence to all PLOS ONE policies on sharing data and materials by including the following statement: "This does not alter our adherence to  PLOS ONE policies on sharing data and materials.” (as detailed online in our guide for authors http://journals.plos.org/plosone/s/competing-interests). If this adherence statement is not accurate and  there are restrictions on sharing of data and/or materials, please state these. Please note that we cannot proceed with consideration of your article until this information has been declared.

3. We note that you have indicated that there are restrictions to data sharing for this study. For studies involving human research participant data or other sensitive data, we encourage authors to share de-identified or anonymized data. However, when data cannot be publicly shared for ethical reasons, we allow authors to make their data sets available upon request. For information on unacceptable data access restrictions, please see http://journals.plos.org/plosone/s/data-availability#loc-unacceptable-data-access-restrictions.

Before we proceed with your manuscript, please address the following prompts:

a) If there are ethical or legal restrictions on sharing a de-identified data set, please explain them in detail (e.g., data contain potentially identifying or sensitive patient information, data are owned by a third-party organization, etc.) and who has imposed them (e.g., a Research Ethics Committee or Institutional Review Board, etc.). Please also provide contact information for a data access committee, ethics committee, or other institutional body to which data requests may be sent.

b) If there are no restrictions, please upload the minimal anonymized data set necessary to replicate your study findings to a stable, public repository and provide us with the relevant URLs, DOIs, or accession numbers. Please see http://www.bmj.com/content/340/bmj.c181.long for guidelines on how to de-identify and prepare clinical data for publication. For a list of recommended repositories, please see https://journals.plos.org/plosone/s/recommended-repositories. You also have the option of uploading the data as Supporting Information files, but we would recommend depositing data directly to a data repository if possible.

Please update your Data Availability statement in the submission form accordingly.

4. In the online submission form you indicate that your data is not available for proprietary reasons and have provided a contact point for accessing this data. Please note that your current contact point is a co-author on this manuscript. According to our Data Policy, the contact point must not be an author on the manuscript and must be an institutional contact, ideally not an individual. Please revise your data statement to a non-author institutional point of contact, such as a data access or ethics committee, and send this to us via return email. Please also include contact information for the third party organization, and please include the full citation of where the data can be found.

5. Please be informed that funding information should not appear in the Acknowledgments section or other areas of your manuscript. We will only publish funding information present in the Funding Statement section of the online submission form. Please remove any funding-related text from the manuscript.

6. If the reviewer comments include a recommendation to cite specific previously published works, please review and evaluate these publications to determine whether they are relevant and should be cited. There is no requirement to cite these works unless the editor has indicated otherwise.

[Note: HTML markup is below. Please do not edit.]

Reviewers' comments:

Reviewer's Responses to Questions

**Comments to the Author**

1. Is the manuscript technically sound, and do the data support the conclusions?

The manuscript must describe a technically sound piece of scientific research with data that supports the conclusions. Experiments must have been conducted rigorously, with appropriate controls, replication, and sample sizes. The conclusions must be drawn appropriately based on the data presented.

Reviewer #1: Yes

Reviewer #2: No

**********

2. Has the statistical analysis been performed appropriately and rigorously?

Reviewer #1: Yes

Reviewer #2: No

**********

3. Have the authors made all data underlying the findings in their manuscript fully available?

The PLOS Data policy requires authors to make all data underlying the findings described in their manuscript fully available without restriction, with rare exception (please refer to the Data Availability Statement in the manuscript PDF file). The data should be provided as part of the manuscript or its supporting information, or deposited to a public repository. For example, in addition to summary statistics, the data points behind means, medians and variance measures should be available. If there are restrictions on publicly sharing data—e.g. participant privacy or use of data from a third party—those must be specified.

Reviewer #1: Yes

Reviewer #2: Yes

**********

4. Is the manuscript presented in an intelligible fashion and written in standard English?

PLOS ONE does not copyedit accepted manuscripts, so the language in submitted articles must be clear, correct, and unambiguous. Any typographical or grammatical errors should be corrected at revision, so please note any specific errors here.

Reviewer #1: Yes

Reviewer #2: Yes

**********

5. Review Comments to the Author

Please use the space provided to explain your answers to the questions above. You may also include additional comments for the author, including concerns about dual publication, research ethics, or publication ethics. (Please upload your review as an attachment if it exceeds 20,000 characters)

Reviewer #1: This manuscript addresses an important but narrow real-world question: how regorafenib is being used in US patients with GIST outside of clinical trials, particularly regarding line of therapy and dosing. The topic is relevant, and the use of a large claims database is appropriate for utilization questions. However, the clinical inferences exceed what the data can support, and there are methodologic, interpretive, and presentation issues that substantially limit impact in its current form.

The manuscript would benefit from tightening its objectives, clarifying what can and cannot be concluded, and reducing causal language. As written, it risks overstating practice-changing implications from descriptive claims data.

Claims-based DOT and TTNT are weak surrogates for clinical benefit and should not be framed as efficacy proxies.

Recommendation:

Reframe the study explicitly as a treatment utilization and sequencing analysis, not an outcomes study. Avoid language implying benefit, tolerance, or superiority.

Overinterpretation of DOT and TTNT: DOT and TTNT are heavily confounded by:

o Physician preference

o Toxicity management strategies

o Access and insurance

o Planned treatment breaks

Statements suggesting longer DOT reflects better tolerance or response are speculative and unsupported.

Recommendation: Replace with neutral phrasing:

“Differences in DOT may reflect prescribing patterns, patient selection, or administrative factors rather than treatment effectiveness.”

Line of therapy classification is fundamentally incomplete and not reflect access to drugs through patient assistance programs.

This limitation should be moved from a minor limitation to a central interpretive caveat and reiterated in the Discussion conclusions.

The following statements are overreaching:

Despite guideline recommendations as an established third-line therapy, regorafenib may be used in earlier lines in the real world, perhaps for its ability to target a wide range of cancer pathways.- there is no data to substantiate this claim for the real world- and this may be misleading.

Discussion statement- The earlier use of regorafenib may be due to physician preference or patient- or market-specific drivers, such as mutation profiles, including KIT activation loop mutations (exon 17 or 18) or SDH deficient GIST which are more responsive to regorafenib and resistant to sunitinib- is completely unfounded with no data.

Does this comment truly represent the time period of the data or is this relevant to today?- potential inaccessibility of sunitinib for some patients, as it is now off patent and does not have any available financial assistance programs.

Reviewer #2: Line 88-89: "This lack of available data highlights a potential practice gap". This sentence is unclear. Perhaps the authors meant to say "The lack of available data limits our ability to determine practice patterns," or similar?

Line 107: Note "error" RE reference. Several other instances in manuscript.

Line 128: CCI score; to simplify for the reader, could you add what the categories mean by including something like: "Briefly, lower CCI score indicates fewer serious comorbidities..." and consider specifying why you split into cohorts of score 2-3 vs score 4+ (from table 1).

Line 138-139: Table 1 subgroups and stratification should line up with the categories listed here. Rather than putting "ie" I suggest detailing exactly what your subgroups are. Keep this consistent with text and figures. In the table for example, "pazopanib" is a header and it is not clear if this means pazopanib only vs any exposure to pazopanib. Further, since ripretinib was FDA approved during the study period (and since it is also an activation loop inhibitor, I suggest adding it as a subgroup. I also noted that sorafenib is listed in table 1, but here, avapritinib is listed. Finally, a big concern about these data is that many of the patients included in the study may have accessed TKI with patient assistance programs or as participants on clinical trials. I assume these data cannot capture this. Perhaps one sensitivity analysis could exclude any patients with a >3 month gap in therapies since we know that most patients with GIST are continuously on TKI therapy. This would hopefully remove any patients who were exposed to unknown TKI therapies.

Line 142: "Groups were also stratified by initial regorafenib dose..." Some clinicians start TKI at a lower dose and escalate as tolerated, and the initial RX may reflect this strategy. Would it be possible to analyze the dose that the patient received most during their time on treatment (acknowledging that some may be at a lower dose due to toxicity).

Line 172: how were the remaining cases categorized (the ones included do not add up to 100%)?

Table 1: typo in header "measured at any point in before index date period". Remove "in"?

Line 187: include “n” for each cohort in body of text (prior IM or SU vs IM+SU)

Line 190: Sentence fragment, please revise. Also include “n” for each cohort (LD vs RSD).

Line 205-206: Without clinical context, I do not think including that 3 patients underwent surgery adds any information. Consider removing.

Line 211-216: include “n” for each cohort (prior IM or SU vs IM+SU; LD vs RSD).

Line 217: Remove “slightly shorter”. These numbers are essentially the same so I suggest leaving out that description. You can just report the median TTNT for each cohort.

Line 221-226: The opening sentence is correct- although I still question whether it is clinically meaningful- but the second sentence RE LD vs RSD is overstated in my opinion. 103 vs 94.5 days are so similar and to highlight that patients on LD have longer DOT seems misleading.

Line 239-242: I suggest commenting on whether result is statistically significant and clinically meaningful to warrant additional evaluation. Further, therapies used in earlier lines often have better clinical outcomes than when used in later lines, yet they may not be better than the approved earlier-line therapies.

Line 245-246: Again, I suggest softening language here and mentioning that difference is similar and not clinically meaningful (1.5 weeks different).

Line 248: I did not see underrepresented status indicated in Table 1. Please define what is meant here.

Line 278-288: You may also mention that treatments received on clinical trials are not captured in these data.

Line 290-291: I think given the limitations of this data type, it is challenging to make any conclusions about utilization patterns.

Line 292-294: As mentioned many times, 1.5 weeks difference should not be highlighted as a true difference. Please revise.

Line 294-295: I’d suggest including more nuance if there is really a case for additional studies. Do you suggest a randomized trial evaluating regorafenib vs sunitinib, or bezuclastinib/sunitinib in 2nd line? Or regorafenib vs ripretinib for activation loop secondary mutations? This does not seem feasible and I’m not sure what additional studies should be done here.

Line 295-297: be sure to include US population; studies exist in other countries.

Line 297-298: I disagree with the conclusion that this adds nuance to the understanding of regorafenib treatment patterns and clinical outcomes. I am also not sure what “insufficiently characterized patient population” means.

**********

6. PLOS authors have the option to publish the peer review history of their article (what does this mean?). If published, this will include your full peer review and any attached files.

If you choose “no”, your identity will remain anonymous but your review may still be made public.

**Do you want your identity to be public for this peer review?**  For information about this choice, including consent withdrawal, please see our Privacy Policy.

Reviewer #1: No

Reviewer #2: No

**********

[NOTE: If reviewer comments were submitted as an attachment file, they will be attached to this email and accessible via the submission site. Please log into your account, locate the manuscript record, and check for the action link "View Attachments". If this link does not appear, there are no attachment files.]

To ensure your figures meet our technical requirements, please review our figure guidelines: https://journals.plos.org/plosone/s/figures

You may also use PLOS’s free figure tool, NAAS, to help you prepare publication quality figures: https://journals.plos.org/plosone/s/figures#loc-tools-for-figure-preparation.

NAAS will assess whether your figures meet our technical requirements by comparing each figure against our figure specifications.

# Author comment: Author response to Decision Letter 1

**DOI:** 10.1371/journal.pone.0353357.r002

30 Mar 2026

Reviewer #1

Clinical inferences exceed what the data can support, and there are methodologic, interpretive, and presentation issues that substantially limit impact in its current form.

The manuscript would benefit from tightening its objectives, clarifying what can and cannot be concluded, and reducing causal language. As written, it risks overstating practice-changing implications from descriptive claims data.

Claims-based DOT and TTNT are weak surrogates for clinical benefit and should not be framed as efficacy proxies.

Thank you for your comments. By addressing the edits and comments from both reviewers, we believe we have addressed this comment.

Reframe the study explicitly as a treatment utilization and sequencing analysis, not an outcomes study. Avoid language implying benefit, tolerance, or superiority.

Overinterpretation of DOT and TTNT: DOT and TTNT are heavily confounded by:

o Physician preference

o Toxicity management strategies

o Access and insurance

o Planned treatment breaks

Statements suggesting longer DOT reflects better tolerance or response are speculative and unsupported.

Replace with neutral phrasing:

“Differences in DOT may reflect prescribing patterns, patient selection, or administrative factors rather than treatment effectiveness.

We have updated the manuscript to include these limitations within the discussion section, limitations section, and conclusions section.

Line of therapy classification is fundamentally incomplete and not reflect access to drugs through patient assistance programs.

This limitation should be moved from a minor limitation to a central interpretive caveat and reiterated in the Discussion conclusions.

We have added the following sentence to the first paragraph in the discussion:

One caveat to this study is the lack of captured use of treatments through clinical trials or patient assistance programs, which are inherent limitations in claims-based studies and limit applicability of results.

We have added the following sentence in the limitations: Additionally, treatments received in clinical trials or through patient assistance programs were not captured in the claims data.

Updated relevant conclusion sentences as follows:

While acknowledging the limitations of a claims-based analysis, this real-world study reveals that the utilization patterns of regorafenib may not always align with guideline recommendations, potentially reflecting use in earlier lines of therapy for a small subset and different dosing.

Despite the noted limitations, this is, to our knowledge, the first study that captures the real-world utilization of regorafenib in advanced or metastatic GIST in a US population.

The following statements are overreaching:

Despite guideline recommendations as an established third-line therapy, regorafenib may be used in earlier lines in the real world, perhaps for its ability to target a wide range of cancer pathways.- there is no data to substantiate this claim for the real world- and this may be misleading.

Discussion statement- The earlier use of regorafenib may be due to physician preference or patient- or market-specific drivers, such as mutation profiles, including KIT activation loop mutations (exon 17 or 18) or SDH deficient GIST which are more responsive to regorafenib and resistant to sunitinib- is completely unfounded with no data.

Updated both statements:

Despite guideline recommendations as an established third-line therapy, anecdotal reports suggest regorafenib may be used in earlier lines of therapy in the real world.[20]

The earlier use of regorafenib may be due to physician preference or patient- or market-specific drivers, such as mutation profiles,[26] or potential inaccessibility of sunitinib for some patients, as it is now off patent and does not have any available financial assistance programs.[27]

Does this comment truly represent the time period of the data or is this relevant to today?- potential inaccessibility of sunitinib for some patients, as it is now off patent and does not have any available financial assistance programs.

We would like to clarify that this statement is meant to align with the study’s observation period, which includes data through 2023, and is not intended to reflect an older era of practice.

We also recognize that access dynamics can evolve over time. While sunitinib is now off patent, some patients may still experience access barriers (e.g., coverage variability, formulary restrictions, or limited assistance options for certain products), which may affect real-world availability. In that context, we felt it was appropriate to note that regorafenib could represent an option to help address potential unmet need in earlier lines of care when access to sunitinib is challenging.

Reviewer #2

Line 88-89: "This lack of available data highlights a potential practice gap". This sentence is unclear. Perhaps the authors meant to say "The lack of available data limits our ability to determine practice patterns," or similar?

Agreed, updated sentence: Thes lack of available data limits the ability to determine practice patterns.

Line 107: Note "error" RE reference. Several other instances in manuscript.

Thank you for letting us know. The navigation does not show those errors in our copy, but we removed navigation links anyway assuming the formatting will be changed for final publication, so they aren’t needed.

Line 128: CCI score; to simplify for the reader, could you add what the categories mean by including something like: "Briefly, lower CCI score indicates fewer serious comorbidities..." and consider specifying why you split into cohorts of score 2-3 vs score 4+ (from table 1).

We have added the following footnote to Table 1: The CCI is a weighted index utilizing the presence of comorbid conditions to predict the risk of death. Lower scores indicate fewer serious comorbidities. Categories were chosen to represent common cutoffs that would impact treatment decisions.

Additionally, the CCI group summary has been updated to a more balanced grouping center around the median.

Line 138-139: Table 1 subgroups and stratification should line up with the categories listed here. Rather than putting "ie" I suggest detailing exactly what your subgroups are. Keep this consistent with text and figures. In the table for example, "pazopanib" is a header and it is not clear if this means pazopanib only vs any exposure to pazopanib. Further, since ripretinib was FDA approved during the study period (and since it is also an activation loop inhibitor, I suggest adding it as a subgroup. I also noted that sorafenib is listed in table 1, but here, avapritinib is listed.

We have updated the text to clearly state the subgroups and removed use of ie. We have also updated the headings of table 1 to match the text.

We have added the following footnote to Table 1:

Note: Baseline demographics for subgroups with n<10 are not presented as statistics from such low patient count subgroups are not expected to be reliable.

Finally, a big concern about these data is that many of the patients included in the study may have accessed TKI with patient assistance programs or as participants on clinical trials. I assume these data cannot capture this. Perhaps one sensitivity analysis could exclude any patients with a >3 month gap in therapies since we know that most patients with GIST are continuously on TKI therapy. This would hopefully remove any patients who were exposed to unknown TKI therapies.

Unfortunately, we are unable to complete additional analyses at this time.

We have added the following sentence to the first paragraph in the discussion:

One caveat to this study is the lack of captured use of treatments through clinical trials or patient assistance programs, which are inherent limitations in claims-based studies and limit applicability of results.

We have also added the following sentence in the limitations: Additionally, treatments received in clinical trials or through patient assistance programs were not captured in the claims data.

Line 142: "Groups were also stratified by initial regorafenib dose..." Some clinicians start TKI at a lower dose and escalate as tolerated, and the initial RX may reflect this strategy. Would it be possible to analyze the dose that the patient received most during their time on treatment (acknowledging that some may be at a lower dose due to toxicity).

This is an interesting perspective and we agree. This still highlights a difference from dose per label. Unfortunately, calculating dose received during entire treatment duration is outside the scope of our current paper.

Line 172: how were the remaining cases categorized (the ones included do not add up to 100%)?

The text was updated to specify subgroups of interest are reported and a footnote has been added under Table 1 to explain which subgroups are reported.

Table 1: typo in header "measured at any point in before index date period". Remove "in"? Updated, thank you

Line 187: include “n” for each cohort in body of text (prior IM or SU vs IM+SU) Added n’s

Line 190: Sentence fragment, please revise. Also include “n” for each cohort (LD vs RSD). Added n’s

Line 205-206: Without clinical context, I do not think including that 3 patients underwent surgery adds any information. Consider removing. Removed sentence

Line 211-216: include “n” for each cohort (prior IM or SU vs IM+SU; LD vs RSD). Added n’s

Line 217: Remove “slightly shorter”. These numbers are essentially the same so I suggest leaving out that description. You can just report the median TTNT for each cohort. Updated to suggested text.

Line 221-226: The opening sentence is correct- although I still question whether it is clinically meaningful- but the second sentence RE LD vs RSD is overstated in my opinion. 103 vs 94.5 days are so similar and to highlight that patients on LD have longer DOT seems misleading. We have updated the text to the following: While numerically different, no clinically meaningful differences were observed in DOT or TTNT between patients on LD regorafenib and those on RSD.

Line 239-242: I suggest commenting on whether result is statistically significant and clinically meaningful to warrant additional evaluation. Further, therapies used in earlier lines often have better clinical outcomes than when used in later lines, yet they may not be better than the approved earlier-line therapies. We have softened this language as follows: DOT was numerically longer in patients who received regorafenib after imatinib or sunitinib only compared to those who received both imatinib and sunitinib; there are multiple potential drivers of longer DOT with earlier use of regorafenib such as specific GIST molecular profiles and improved patient tolerance prior to multiple lines of therapy. Similarly, there may be drivers of shorter DOT in those who received regorafenib after two therapies such as a more aggressive or unresponsive disease profile.

Line 245-246: Again, I suggest softening language here and mentioning that difference is similar and not clinically meaningful (1.5 weeks different). Edited to: Median DOT and TTNT were similar between patients who received LD vs those who received RSD.

Line 248: I did not see underrepresented status indicated in Table 1. Please define what is meant here. Edited to “underrepresented” as follows: “While limited by several factors noted below, these results help portray the real-world treatment patterns and characteristics of a patient population with advanced metastatic GIST.

Line 278-288: You may also mention that treatments received on clinical trials are not captured in these data. Added sentence “Additionally, treatments received in clinical trials were not captured in the claims data.”

Line 290-291: I think given the limitations of this data type, it is challenging to make any conclusions about utilization patterns. Updated line to soften this sentence: While acknowledging its limitations, this real-world study reveals that the utilization patterns of regorafenib may not always align with guideline recommendations, potentially reflecting use in earlier lines of therapy for a small subset and different dosing.

Line 292-294: As mentioned many times, 1.5 weeks difference should not be highlighted as a true difference. Please revise. Revised to phrase DOT as similar instead of different: ”Patients on LD regorafenib had DOT and TTNT similar to those on RSD.”

Line 294-295: I’d suggest including more nuance if there is really a case for additional studies. Do you suggest a randomized trial evaluating regorafenib vs sunitinib, or bezuclastinib/sunitinib in 2nd line? Or regorafenib vs ripretinib for activation loop secondary mutations? This does not seem feasible and I’m not sure what additional studies should be done here. Revised to: However, additional studies will be needed to clarify benefits in earlier therapy lines and, as newer treatments emerge, to understand responses across molecular profiles so that the most effective drugs can be sequenced earlier to match each patient’s profile.

Line 295-297: be sure to include US population; studies exist in other countries. Added “US population” at the end of the sentence:

To our knowledge, this is the first study that captures the real-world utilization of regorafenib in advanced or metastatic GIST in a US population.

Line 297-298: I disagree with the conclusion that this adds nuance to the understanding of regorafenib treatment patterns and clinical outcomes. I am also not sure what “insufficiently characterized patient population” means. We have updated the final sentence to state: Findings from this study help portray the real-world treatment patterns and characteristics of a patient population with advanced or metastatic disease.

Attachment Submitted filename: Journal revision table_18MAR26_Final.docx

# Peer review: Decision Letter 1

**DOI:** 10.1371/journal.pone.0353357.r003

**Authors:** Anette Duensing, Anette Duensing

14 Apr 2026

PONE-D-25-61423R1

Real-world regorafenib use among patients with advanced gastrointestinal stromal tumor in the United States

PLOS One

Dear Dr. Denu,

Thank you for submitting your manuscript to PLOS ONE. After careful consideration, we feel that the manuscript has improved but some small issues remain. We invite you to submit a revised version of the manuscript that addresses the points raised during this second review process.

Please submit your revised manuscript by May 29 2026 11:59PM. If you will need more time than this to complete your revisions, please reply to this message or contact the journal office at plosone@plos.org. When you're ready to submit your revision, log on to https://www.editorialmanager.com/pone/ and select the 'Submissions Needing Revision' folder to locate your manuscript file.

Please include the following items when submitting your revised manuscript:

- A letter that responds to each point raised by the academic editor and reviewer(s). You should upload this letter as a separate file labeled 'Response to Reviewers'.
- A marked-up copy of your manuscript that highlights changes made to the original version. You should upload this as a separate file labeled 'Revised Manuscript with Track Changes'.
- An unmarked version of your revised paper without tracked changes. You should upload this as a separate file labeled 'Manuscript'.

If you would like to make changes to your financial disclosure, please include your updated statement in your cover letter. Guidelines for resubmitting your figure files are available below the reviewer comments at the end of this letter.

If applicable, we recommend that you deposit your laboratory protocols in protocols.io to enhance the reproducibility of your results. Protocols.io assigns your protocol its own identifier (DOI) so that it can be cited independently in the future. For instructions see: https://journals.plos.org/plosone/s/submission-guidelines#loc-laboratory-protocols. Additionally, PLOS ONE offers an option for publishing peer-reviewed Lab Protocol articles, which describe protocols hosted on protocols.io. Read more information on sharing protocols at https://plos.org/protocols?utm_medium=editorial-email&utm_source=authorletters&utm_campaign=protocols.

As the corresponding author, your ORCID iD is verified in the submission system and will appear in the published article. PLOS supports the use of ORCID, and we encourage all coauthors to register for an ORCID iD and use it as well. Please encourage your coauthors to verify their ORCID iD within the submission system before final acceptance, as unverified ORCID iDs will not appear in the published article. *Only*  the individual author can complete the verification step; PLOS staff *cannot*  verify ORCID iDs on behalf of authors.

We look forward to receiving your revised manuscript.

Kind regards,

Anette Duensing, M.D.

Academic Editor

PLOS One

Journal Requirements:

1. If the reviewer comments include a recommendation to cite specific previously published works, please review and evaluate these publications to determine whether they are relevant and should be cited. There is no requirement to cite these works unless the editor has indicated otherwise.

2. Please review your reference list to ensure that it is complete and correct. If you have cited papers that have been retracted, please include the rationale for doing so in the manuscript text, or remove these references and replace them with relevant current references. Any changes to the reference list should be mentioned in the rebuttal letter that accompanies your revised manuscript. If you need to cite a retracted article, indicate the article’s retracted status in the References list and also include a citation and full reference for the retraction notice.

[Note: HTML markup is below. Please do not edit.]

Reviewer's Responses to Questions

**Comments to the Author**

1. If the authors have adequately addressed your comments raised in a previous round of review and you feel that this manuscript is now acceptable for publication, you may indicate that here to bypass the “Comments to the Author” section, enter your conflict of interest statement in the “Confidential to Editor” section, and submit your "Accept" recommendation.

Reviewer #1: All comments have been addressed

Reviewer #2: (No Response)

**********

2. Is the manuscript technically sound, and do the data support the conclusions?

The manuscript must describe a technically sound piece of scientific research with data that supports the conclusions. Experiments must have been conducted rigorously, with appropriate controls, replication, and sample sizes. The conclusions must be drawn appropriately based on the data presented.

Reviewer #1: Yes

Reviewer #2: Partly

**********

3. Has the statistical analysis been performed appropriately and rigorously?

Reviewer #1: Yes

Reviewer #2: I Don't Know

**********

4. Have the authors made all data underlying the findings in their manuscript fully available?

The PLOS Data policy requires authors to make all data underlying the findings described in their manuscript fully available without restriction, with rare exception (please refer to the Data Availability Statement in the manuscript PDF file). The data should be provided as part of the manuscript or its supporting information, or deposited to a public repository. For example, in addition to summary statistics, the data points behind means, medians and variance measures should be available. If there are restrictions on publicly sharing data—e.g. participant privacy or use of data from a third party—those must be specified.

Reviewer #1: Yes

Reviewer #2: Yes

**********

5. Is the manuscript presented in an intelligible fashion and written in standard English?

PLOS ONE does not copyedit accepted manuscripts, so the language in submitted articles must be clear, correct, and unambiguous. Any typographical or grammatical errors should be corrected at revision, so please note any specific errors here.

Reviewer #1: Yes

Reviewer #2: Yes

**********

6. Review Comments to the Author

Please use the space provided to explain your answers to the questions above. You may also include additional comments for the author, including concerns about dual publication, research ethics, or publication ethics. (Please upload your review as an attachment if it exceeds 20,000 characters)

Reviewer #1: All comments appreciated. No further recommendations.

Appreciate the time and effort to make the changes.

Reviewer #2: Please update the abstract, (line 59) first sentence in the conclusion to conclude that TTNT *and* DOT were similar for LD and RSD.

Please correct the sentence in the abstract (line 54): "Patients who received prior imatinib or sunitinib alone

54 before regorafenib reported a numerically longer median DOT". ...The patients did not "report". You can say, for example, "The data showed that patients who received...had a numerically longer median DOT."

Please the language for the sentence in the abstract (line 56): "LD demonstrated a longer median DOT compared to RSD" and instead indicate that they are similar.

**********

7. PLOS authors have the option to publish the peer review history of their article (what does this mean?). If published, this will include your full peer review and any attached files.

If you choose “no”, your identity will remain anonymous but your review may still be made public.

**Do you want your identity to be public for this peer review?**  For information about this choice, including consent withdrawal, please see our Privacy Policy.

Reviewer #1: No

Reviewer #2: No

**********

[NOTE: If reviewer comments were submitted as an attachment file, they will be attached to this email and accessible via the submission site. Please log into your account, locate the manuscript record, and check for the action link "View Attachments". If this link does not appear, there are no attachment files.]

To ensure your figures meet our technical requirements, please review our figure guidelines: https://journals.plos.org/plosone/s/figures

You may also use PLOS’s free figure tool, NAAS, to help you prepare publication quality figures: https://journals.plos.org/plosone/s/figures#loc-tools-for-figure-preparation.

NAAS will assess whether your figures meet our technical requirements by comparing each figure against our figure specifications.

# Author comment: Author response to Decision Letter 2

**DOI:** 10.1371/journal.pone.0353357.r004

2 Jun 2026

Reviewer #1: All comments appreciated. No further recommendations.

Appreciate the time and effort to make the changes.

We thank the reviewer for their positive feedback and effort to help make our manuscript suitable for publication.

Reviewer #2: Please update the abstract, (line 59) first sentence in the conclusion to conclude that TTNT *and* DOT were similar for LD and RSD.

This has been corrected and now reads: “Patients on LD and RSD had similar DOT and TTNT.”

Please correct the sentence in the abstract (line 54): "Patients who received prior imatinib or sunitinib alone

54 before regorafenib reported a numerically longer median DOT". ...The patients did not "report". You can say, for example, "The data showed that patients who received...had a numerically longer median DOT."

This has been corrected and now reads: “Patients who received prior imatinib or sunitinib alone before regorafenib had a numerically longer median DOT…”

Please the language for the sentence in the abstract (line 56): "LD demonstrated a longer median DOT compared to RSD" and instead indicate that they are similar.

This has been corrected and now reads: “Patients receiving LD and RSD demonstrated similar median DOT…and TTNT…”

Attachment Submitted filename: Response to Reviews 4.30.26.docx

# Editor report: Decision Letter 2

**DOI:** 10.1371/journal.pone.0353357.r005

**Authors:** Anette Duensing, Anette Duensing, Anette Duensing

24 Jun 2026

Real-world regorafenib use among patients with advanced gastrointestinal stromal tumor in the United States

PONE-D-25-61423R2

Dear Dr. Denu,

We’re pleased to inform you that your manuscript has been judged scientifically suitable for publication and will be formally accepted for publication once it meets all outstanding technical requirements.

Within one week, you’ll receive an e-mail detailing the required amendments. When these have been addressed, you’ll receive a formal acceptance letter and your manuscript will be scheduled for publication.

An invoice will be generated when your article is formally accepted. Please note, if your institution has a publishing partnership with PLOS and your article meets the relevant criteria, all or part of your publication costs will be covered. Please make sure your user information is up-to-date by logging into Editorial Manager at Editorial Manager® and clicking the ‘Update My Information' link at the top of the page. For questions related to billing, please contact billing support.

If your institution or institutions have a press office, please notify them about your upcoming paper to help maximize its impact. If they’ll be preparing press materials, please inform our press team as soon as possible -- no later than 48 hours after receiving the formal acceptance. Your manuscript will remain under strict press embargo until 2 pm Eastern Time on the date of publication. For more information, please contact onepress@plos.org.

Kind regards,

Anette Duensing, M.D.

Academic Editor

PLOS One

Additional Editor Comments (optional):

Reviewers' comments:

# Editor report: Acceptance letter

**DOI:** 10.1371/journal.pone.0353357.r006

**Authors:** Anette Duensing, Anette Duensing, Anette Duensing

PONE-D-25-61423R2

PLOS One

Dear Dr. Denu,

I'm pleased to inform you that your manuscript has been deemed suitable for publication in PLOS One. Congratulations! Your manuscript is now being handed over to our production team.

At this stage, our production department will prepare your paper for publication. This includes ensuring the following:

* All references, tables, and figures are properly cited

* All relevant supporting information is included in the manuscript submission,

* There are no issues that prevent the paper from being properly typeset

You will receive further instructions from the production team, including instructions on how to review your proof when it is ready. Please keep in mind that we are working through a large volume of accepted articles, so please give us a few days to review your paper and let you know the next and final steps.

Lastly, if your institution or institutions have a press office, please let them know about your upcoming paper now to help maximize its impact. If they'll be preparing press materials, please inform our press team within the next 48 hours. Your manuscript will remain under strict press embargo until 2 pm Eastern Time on the date of publication. For more information, please contact onepress@plos.org.

You will receive an invoice from PLOS for your publication fee after your manuscript has reached the completed accept phase. If you receive an email requesting payment before acceptance or for any other service, this may be a phishing scheme. Learn how to identify phishing emails and protect your accounts at https://explore.plos.org/phishing.

If we can help with anything else, please email us at customercare@plos.org.

Thank you for submitting your work to PLOS ONE and supporting open access.

Kind regards,

PLOS ONE Editorial Office Staff

on behalf of

Dr Anette Duensing

Academic Editor

PLOS One
